# *In vitro* comparison of the surface microhardness of three nanohybrid resin composites immersed in different pigmented beverages for varying exposure times

**DOI:** 10.4317/jced.62640

**Published:** 2025-05-01

**Authors:** Wendy Ayala-Amaya, Leonor Castro-Ramirez, Flor Santander-Rengifo, María Alvino-Vales, Denisse Turpo-Claudio, César Cayo-Rojas

**Affiliations:** 1 Universidad Privada San Juan Bautista, School of Stomatology, Lima, Peru

## Abstract

**Background:**

To assess the surface microhardness (SMH) of three nanohybrid resin composites after immersion in different pigmented beverages for varying exposure times.

**Material and Methods:**

This *in vitro* longitudinal experimental study consisted of 120 samples equally distributed for Filtek Z350XT, Tetric N-Ceram, and Brillant NG resin composites. These were immersed for 1, 4, and 7 days in burgundy wine, ground coffee, purple corn beverage, and artificial saliva (control). For statistical analysis, the Kruskal-Wallis H-test and Friedman’s test with Bonferroni’s post hoc test were used. The significance level was *p*<0.05.

**Results:**

When comparing the SMH on days 1, 4, and 7 of immersion in different beverages, it was evident that there were no significant differences in the composite resins Filtek Z350XT (*p* = 0.678, *p* = 0.731, and p*p* = 0.225; respectively) and Tetric N-Ceram (*p* = 0.214, *p* = 0.382, and *p* = 0.521; respectively). However, there were significant differences in Brilliant NG resin composite (*p* = 0.010, *p* = 0.011, and *p* = 0.006; respectively), showing that coffee significantly decreased the SMH of this resin compared to artificial saliva (*p* = 0.012, *p* = 0.007, and *p* = 0.004; respectively). In addition, burgundy wine, ground coffee, and purple corn significantly decreased the SMH of the three composite resins over time (*p*<0.001).

**Conclusions:**

Brilliant NG resin composite significantly decreased its SMH on days 1, 4 and 7 of immersion in all beverages compared to Filtek Z350XT and Tetric N-Ceram resin composites. On the other hand, over time, burgundy wine, ground coffee, and purple corn significantly decreased the SMH of all three resin composites, while artificial saliva did not.

** Key words:**Microhardness, nanohybrid resins, pigment drinks, in vitro study.

## Introduction

Currently, resin composite is the material of choice for restoring a tooth (1,2). Therefore, over time, advances in the chemical composition of the material have led to modifications in order to obtain better mechanical and aesthetic properties (3). However, despite this, the resin is still susceptible to softening by organic acids and various food and beverage components (4).

Beverages, food, and the oral environment, including saliva and pH, affect the mechanical properties, which can lead to changes in the wear resistance of the resin composite and a reduction in surface hardness (5).

Microhardness is defined as the resistance to permanent penetration or indentation (6,7), being an indicator of resistance to deformation, and is determined by dividing the strength by the indented surface area (6). A composite resin with lower microhardness is more susceptible to scratches and surface defects that ultimately reduce the flexural strength of the materials and lead to premature failure of the restoration (8). The Vickers test is one of the most commonly used tests to evaluate this property and has been employed in several studies (6,7).

Dark beverages have been reported to alter the color of teeth and restorative materials, including coffee, tea, red wine, and some carbonated beverages (9,10). Purple corn beverage is one of the most consumed beverages in some Latin American countries, and its preparation may include sugar, lemon, cinnamon, and various fruits, which could affect the surface of resin composite due to its acidic pH (9). Therefore, it is important to investigate the mechanical properties of the most commonly used resin composites on the market to provide evidence that will allow the dental professional to make decisions that guarantee the clinical success of the restorative treatment in the long term, as previous studies have shown that coffee, burgundy wine, and purple corn are beverages that alter the color stability of nanohybrid resin composites (11-13).

Therefore, the aim of the present investigation was to assess the SMH of three nanohybrid resin composites after immersion in different pigmented beverages for varying exposure times. The null hypothesis was that there is no significant difference when comparing the SMH of three nanohybrid resin composites after immersion in different pigmented beverages for varying exposure times.

## Material and Methods

1. Study design

The design of this research was experimental *in vitro* and longitudinal. This study was conducted at the High Technology Laboratory Certificate (ISO/IEC Standard: 17025), Lima, Peru, between February and May 2024. The Institutional Research Ethics Committee of the Universidad Privada San Juan Bautista (UPSJB) approved the execution of this study with Letter No. 1648-2023-CIEI-UPSJB on November 4, 2023. In addition, this study considered the CRIS guideline (Checklist for Reporting In-vitro Studies) (14).

2. Calculation and sample selection

**Figure 1:**
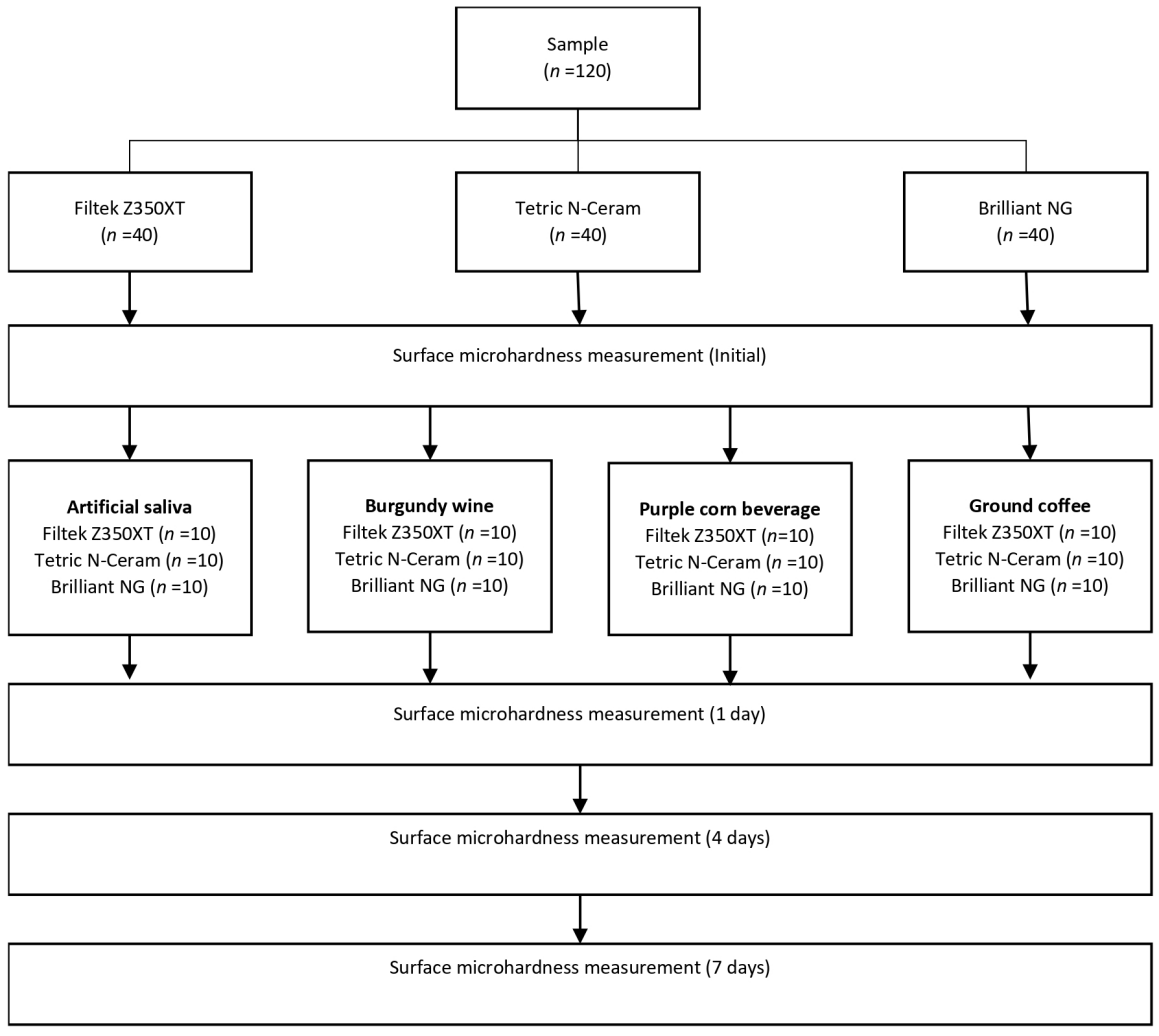
Distribution of groups, according to sample size.

The minimum total sample size (n = 120) was calculated based on data obtained in a previous pilot study with 5 sample units per group, where the formula for analysis of variance with 4 repeated measures was applied in the statistical software G*Power version 3.1.9.7, considering a significance level (α) = 0.05 and a statistical power (1-β) = 0.80, with an effect size of 0.32. The composite resin discs were randomly divided to form the groups (n = 10) to be immersed in artificial saliva, burgundy wine, purple corn, and ground coffee (Fig. 1).

3. Characteristics and sample preparation

Three resin composites were used to produce the samples: Filtek Z350XT (3M, ESPE, St. Paul, MN, USA), Tetric N-Ceram (Ivoclar Vivadent, Schaan, Liechtenstein), and Brilliant NG (Coltene/Whaledent AG, Altstätten, Switzerland) ( Table 1).

Forty discs of each type of resin were made up for a total of 120 units of analysis. The resin composite was placed in 2 mm increments in a cylindrical steel mold measuring 5 mm in diameter and 5 mm in height, and celluloid tape was applied to both sides and gently pressed with a 1 mm thick microscope slide to remove excess material (7,15,16). Finally, all samples were light-cured with an LED lamp (Woodpecker® LM-1, Woodpecker, Guilin, Guangxi, China) at 1200 mW/cm² for 20 seconds. The intensity of the light curing unit was checked with a radiometer (Woodpecker® LM-1, Woodpecker, Guilin, Guangxi, China). The same operator polished each disc for 20 s using a four-stage disc system (Sof-Lex, 3M/ESPE), following the manufacturer’s instructions. The processed samples were checked for voids, cracks, and irregularities. All discs were placed in an airtight container filled with distilled water and kept at 37°C for 24 h (15,16).

4. Immersion protocol

Ten discs of each composite resin were immersed in each of the solutions ( Table 2): 50 mL of artificial saliva, 50 mL of burgundy wine, 50 mL of purple corn and 50 mL of ground coffee.

Artificial saliva and burgundy wine were used directly without any preparation; coffee was prepared with 25 g in 250 mL of hot water (15), and chicha morada was prepared with two purple cobs per half liter of water, boiled for 10 minutes (9), and sugar, lemon, pineapple, and apple were added. All immersion media were placed in beakers and covered during the experiment to prevent evaporation of the solutions. All beverages were used at their usual drinking temperature (artificial saliva ± 37°C, ground coffee ± 70°C, burgundy wine ± 10°C, and purple corn ± 10°C) (17). The immersion times used were 1, 4, and 7 days.

5. Surface microhardness test

This was done before and after the immersion phase. Each sample group was dried with a paper towel to proceed with the measurement. The microhardness of each sample group was measured using an ‘Electronic Vickers Microhardness Tester’ (LG-HV 1000) (LG Electronics, Seoul, Korea). A loading force of 100 grams was used for 10 seconds to the nearest 1 µm - 40X. With a total of 3 indentations on the surface of the resin composite discs, the results of the Vickers microhardness measurements were averaged to obtain a single value per sample.

6. Statistical analysis

SPSS (Statistical Package for the Social Sciences Inc., IBM, NY, USA) software version 28.0 was used for data analysis. Descriptive statistics were calculated for the mean, median, and interquartile range. To test the normality and homoscedasticity of the data, the Shapiro-Wilk test and Levene’s test were used, respectively. The results indicated that the data were not normally distributed. Consequently, the Kruskal-Wallis non-parametric H-test for three independent measures at each of the dive times was considered the most appropriate statistical method. Furthermore, to compare more than two related measures, the Friedman test was used. In addition, Bonferroni’s post hoc was used to refine the statistical differences, depending on the results obtained in the Kruskal-Wallis and Friedman tests. Statistical significance was set at *p*<0.05.

## Results

Before immersion, it could be observed that there were no significant differences in DM when comparing the subgroups within the Filtek Z350XT (*p* = 0.390), Tetric N-Ceram (*p* = 0.171), and Brilliant NG resin composites (*p* = 0.103), which evidenced that before the experimental test the resin blocks were standardized ( Table 3).

On the other hand, when comparing the SMH on day 1, day 4, and day 7 of immersion in different beverages, it was evident that there were no significant differences in the resin composites Filtek Z350XT (*p* = 0.678, *p* = 0.731, and *p* = 0.225; respectively) and Tetric N-Ceram (*p* = 0.214, *p* = 0.382, and *p* = 0.521; respectively). However, significant differences were observed for Brilliant NG resin composite (*p* = 0.010, *p* = 0.011, and *p*= 0.006; respectively), showing that coffee significantly decreased the SMH of Brilliant NG resin compared to artificial saliva at the same immersion times (*p* = 0.012, *p* = 0.007, and *p* = 0.004; respectively) ( Table 3).

Over time, it was observed that artificial saliva did not significantly affect the SMH of the resin composites, while purple corn, burgundy wine, and coffee significantly affected the SMH of Filtek Z350XT (*p*<0.001), Tetric N-Ceram (*p*<0.001), and Brilliant NG (*p*<0.001) resin composites. As a result, burgundy wine significantly decreased the SMH of Filtek Z350XT and Brilliant NG resins at day 1 of immersion (*p*<0.05 and *p*<0.05, respectively), and these values did not change over time. There was a significant decrease in SMH in the Tetric N-Ceram resin at day 4 of immersion, with these values not varying significantly until day 7 of immersion. In addition, purple chicha and coffee significantly decreased the SMH of Filtek Z350XT, Tetric N-Ceram, and Brilliant NG resins at day 4 of immersion (*p*<0.05, *p*<0.05, and *p*<0.05, respectively), with these values not varying until day 7 of immersion. Finally, at 7 days of immersion, coffee and purple corn significantly decreased the SMH of Tetric N-Ceram resin composite (*p*<0.05 and *p*<0.05, respectively), compared to the values obtained at day 1 of immersion ( Table 3).

## Discussion

SMH is one of the most valued properties of restorative materials, as they must be able to withstand the occlusal forces and potential chemical challenges present in the oral cavity (16). The study’s goal was to find out how different pigmented beverages affected the SMH of Filtek Z350XT, Tetric N-Ceram, and Brillant NG resin composites after 1, 4, and 7 days. As a result, the Filtek Z350XT, Tetric N-Ceram, and Brillant NG resins showed a significant decrease in SMH over time after immersion in ground coffee, burgundy wine, and purple corn, so the null hypothesis had to be rejected.

The findings obtained in the present study showed that coffee significantly decreased the SMH of Brillant NG at all immersion times. This may be due to its composition, since this resin has only Bis-GMA, TEGDMA, and Bis-EMA, which are very susceptible to softening by chemicals (16). Above all, this may be due to the presence of TEGDMA, a hydrophilic monomer that not only creates a denser polymer network but also has the ability to absorb large amounts of water (14). This associated with the temperature of the coffee (+/- 70 °C) may have reduced its SMH, as high immersion temperature has been shown to promote intensive degradation of the resin matrix (17,18). It should be noted that Filtek Z350XT and Tetric N-Ceram were not significantly affected by the coffee, which could be related to the presence of UDMA in their composition; this is because resins with UDMA present a higher reactivity and molecular mobility, producing a higher degree of conversion, thus improving the polymerization, physical, and mechanical properties of the resin composites (19-22). Furthermore, it should be noted that adequate polymerization is critical for the success of resin composite restorations, as incomplete polymerization is responsible for water absorption, which reduces wear resistance and promotes leaching of residual monomers (4).

It was found that burgundy wine significantly decreased the SMH of Filtek Z350XT and Brilliant NG resins at day 1 of immersion. This may be because wine has been reported to affect the colour stability of resin composites (23-25), due to high levels of chromogenic chemicals, such as polyphenols and tannins, which can enter the resin matrix and cause changes in the structure. It has been reported that zirconia/silica fillers are prone to water attack, and the smaller surface area of spherically shaped zirconia/silica fillers bonded to the resin matrix leached more easily (4). In addition, the presence of barium as an electropositive metal filler tends to react with water, whereby hydrogen ions in water replace the spaces occupied by zinc and barium (8), thus affecting the SMH. As the concentration of hydroxyl ions increases, the siloxane (Si-O-Si) bonds in the silica network begin to break down and an autocatalytic cycle of surface degradation occurs, leading to a reduction in microhardness (8,16). It is worth mentioning that Tetric N-Ceram lacks silica or zirconium in its composition, which may have favored that it is not very affected by wine. However, on the 4th day of immersion, its microhardness decreased until the 7th day, due to the presence of barium that reacts with water, thus reducing its MSH (8), but this would occur in the medium term, as one of the factors of microhardness loss is prolonged exposure to acidic beverages (8,26).

Purple corn and coffee, they significantly decreased the SMH of the three resin composites from day 4 of immersion until day 7 of immersion. The purple corn has pigments similar to coffee, called anthocyanins, which, associated with low pH levels in the beverage, can cause damage to the surface of the material and allow the penetration of pigments (9,10,27), causing changes in the structure of the resin composite.

It should be considered that the microhardness of resins is influenced by various parameters, such as the volume/weight of the inorganic particles, the constitution of the polymer matrix, the morphology, size, and distribution of the filler particles, and the quality of the transmitted light (6,28,29); reasons why the effect of different beverages can be variable between the different resin composites used.

In regard to the pH of the beverages, it has been reported that low pH values usually have a negative effect on the surface of composite resins (3,8,30). This study’s resins had different compositions, so microhardness reduction varied even though all beverages had an acidic pH.

The importance of this study lies in the fact that it provides evidence about the behavior of nanohybrid resins when exposed to commonly consumed beverages. Consequently, if the restoration technique is appropriate, the durability over time depends on the resistance of these restorative materials, as this can compromise their functionality and aesthetics, producing premature wear, fractures, cracks, stains, or discoloration. Therefore, the data obtained in this research provides evidence that will allow the dental professional to make decisions that will guarantee the success of the restorative treatment in the long term.

As a strength, it can be mentioned that the design of the study allowed for the demonstration that the resin composite discs presented a standardized surface microhardness at the beginning of the study, as they presented similar values before being immersed. In addition, the microhardness was evaluated with the Vickers test, which, in comparison to the Brinell or Rockwell machines, the Vickers machine is more accurate (16). Another strength is that 7 days of immersion was used, which is equivalent to 3 months of daily consumption (8); furthermore, the beverages used were used at their usual drinking temperature, which would reflect daily conditions (17). Shade A2 was used in this study because it is one of the most common shades in human teeth and is commonly used in clinical practice (31).

Of the limitations, being an *in vitro* study, it cannot simulate a real clinical situation, due to the fact that various substances may be present in the oral environment, such as saliva and food, among others; it should also be considered that the accumulated biofilm may produce acidic substances that can cause surface degradation, leading to softening and roughening of the surface of the material, also compromising the hardness of the resin composites (6,32). Furthermore, it is important to note that the use of a stainless steel metal matrix for sample preparation may underestimate the depth of polymerization that actually occurs in a clinical situation because the inner walls of the metal matrix do not scatter light but absorb it, reducing the number of photons available for activation (33).

It is recommended to evaluate the relationship between microhardness, roughness, and color stability among resin composites exposed to different beverages, as there could be an inverse relationship between the change in microhardness and surface roughness (8). It is also suggested to study the SMH of nanohybrid resin composites immersed in aboriginal natural beverages from other countries with varying pH.

## Conclusions

Brilliant NG resin significantly decreased its SMH on days 1, 4 and 7 of immersion in all pigmented beverages compared to Filtek Z350XT and Tetric N-Ceram resin composites. However, over time, burgundy wine, coffee grounds and purple corn significantly decreased the SMH of all three resin composites, while artificial saliva did not.

## Figures and Tables

**Table 1:** Technical profile of the resin composites used.

Product	Composition	Filling % (% wt/vol) (% weight /vol)	Manufacturer	Lot
Filtek Z350 XT A2	Matrix: Bis-GMA, UDMA, TEGDMA, Bis-EMA Filler: zirconia/silica, barium glass, ytterbium trifluoride, mixed oxide prepolymer	75 wt - 59.5 vol	3M, ESPE, St. Paul, Minnesota, EE. UU.	9993539
Tetric® N-Ceram A2	Matrix: Bis-GMA, Bis-EMA, UDMA Filler: Dimethacrylates, additives, catalysts, stabilizer sand pigments, barium glass, ytterbium trifluoride, mixed oxide and prepolymerized filler	81 wt - 57 vol	Ivoclar Vivadent, Schaan, Liechtenstein.	Z05ST7
Brilliant™ NG A2	Matrix: BisGMA, BisEMA, TEGDMA Filler: Dental glass, amorphous silica	80 wt - 65 vol.	Coltene/Whaledent AG, Altstätten, Suiza	M39476

**Table 2:** Immersion solutions used.

Product	pH	Manufacturer
Artificial saliva	6.75	Laboratorios Unidos, Lima, Peru.
Burgundy wine	3.82	Santiago Queirolo, Ica, Peru.
Purple corn	3.5	----------
Ground coffee	5.45	Cafetal, Chanchamayo, Peru.

pH: potential of hydrogen.

**Table 3:** Comparison of the surface microhardness (VHN) of three resin composites immersed in different pigmented beverages for varying exposure times.

Resin composite	Beverages	n	Before immersion				1 day				4 days				7 days				p**
			Mean	Median	IQR	p*	Mean	Median	IQR	p*	Mean	Median	IQR	p*	Mean	Median	IQR	p*
Filtek Z350XT	Artificial saliva	10	72.60	73.80	4.17	0.390	72.39	73.50	5.80	0.678	71.87	73.00	4.98	0.731	72.11	72.90	4.73	0.225	0.432
	Purple corn	10	72.91	71.55 ^x^	6.30		71.27	69.60 ^x,y^	7.47		70.47	69.70 ^y^	7.92		69.36	69.45 ^y^	9.42		<0.001**
	Burgundy wine	10	74.50	75.60 ^x^	4.15		72.62	73.40 ^y^	5.42		71.91	71.95 ^y^	3.67		72.12	72.35 ^y^	4.05		<0.001**
	Ground coffee	10	74.20	74.55^ x^	3.13		71.54	71.60 ^x,y^	2.83		71.14	71.30 ^y^	2.42		70.64	70.70 ^y^	1.55		<0.001**
Tetric N-Ceram	Artificial saliva	10	47.80	48.85	9.10	0.171	47.48	47.95	7.68	0.214	47.33	48.40	7.93	0.382	46.83	47.45	8.68	0.521	0.080
	Purple corn	10	48.44	47.40 ^x^	3.00		47.43	46.75 ^x,y^	0.83		46.68	45.90 ^y,z^	1.80		46.09	45.30 ^z^	2.65		<0.001**
	Burgundy wine	10	50.67	50.55 ^x^	3.52		49.37	48.70 ^x,y^	3.67		48.45	48.10 ^y^	5.15		47.90	47.80 ^y^	4.20		<0.001**
	Ground coffee	10	51.85	50.40 ^x^	4.03		50.20	48.50 ^x,y^	3.60		49.33	48.25 ^y,z^	3.40		46.96	46.65 ^z^	5.30		<0.001**
Brilliant NG	Artificial saliva	10	63.60	63.45	6.95	0.103	63.19	62.70 ^A^	6.55	0.010*	63.10	62.20 ^A^	7.85	0.011*	63.30	63.10 ^A^	5.88	0.006*	0.183
	Purple corn	10	62.05	62.50 ^x^	6.00		60.64	61.20 ^A,B^^; ^^x,y^	5.80		58.52	59.20 ^A,B^^; y^	6.88		56.98	57.80 ^A,B^^; y^	7.98		<0.001**
	Burgundy wine	10	60.54	59.60 ^x^	6.20		58.05	57.10 ^A,B^^; y^	5.80		57.93	57.25 ^A,B^^; y^	5.53		57.19	56.55 ^A,B^^; y^	6.15		<0.001**
	Ground coffee	10	57.55	56.85 ^x^	13.47		55.02	54.20 ^B; ^^x,y^	10.25		54.59	53.30 ^B; y^	11.35		54.10	52.30 ^B;^ ^y^	11.38		<0.001**

n: sample size; IQR: Interquartile Range; *Based on Kruskal Wallis test (*p*<0.05, significant differences); **Based on Friedman test (*p*<0.05, significant differences); A and B: different letters in each column of the median indicated significant differences (*p*<0.05) based on Bonferroni’s post hoc. x,y,z: different letters in the same row indicated significant differences (*p*<0.05) based on Bonferroni’s post hoc.

## Data Availability

The datasets used and/or analyzed during the current study are available from the corresponding author.
